# Effect of Pain Education and Exercise on Pain and Function in Chronic Achilles Tendinopathy: Protocol for a Double-Blind, Placebo-Controlled Randomized Trial

**DOI:** 10.2196/19111

**Published:** 2020-11-03

**Authors:** Andrew A Post, Ebonie K Rio, Kathleen A Sluka, G Lorimer Moseley, Emine O Bayman, Mederic M Hall, Cesar de Cesar Netto, Jason M Wilken, Jessica F Danielson, Ruth Chimenti

**Affiliations:** 1 Department of Physical Therapy & Rehabilitation Science University of Iowa Iowa City, IA United States; 2 School of Allied Health La Trobe University Bundoora Australia; 3 IMPACT in Health University of South Australia Adelaide Australia; 4 Departments of Biostatistics and Anesthesia University of Iowa Iowa City, IA United States; 5 University of Iowa Sports Medicine Department of Orthopaedics & Rehabilitation University of Iowa Iowa City, IA United States; 6 Department of Orthopaedics & Rehabiliation University of Iowa Iowa City, IA United States; 7 Institute for Clinical and Translation Science University of Iowa Iowa City, IA United States

**Keywords:** Achilles tendon, tendinopathy, rehabilitation, pain, tendon, patient education

## Abstract

**Background:**

Achilles tendinopathy (AT) rehabilitation traditionally includes progressive tendon loading exercises. Recent evidence suggests a biopsychosocial approach that incorporates patient education on psychosocial factors and mechanisms of pain can reduce pain and disability in individuals with chronic pain. This is yet to be examined in individuals with AT.

**Objective:**

This study aims to compare the effects on movement-evoked pain and self-reported function of pain education as part of a biopsychosocial approach with pathoanatomical education for people with AT when combined with a progressive tendon loading exercise program.

**Methods:**

A single-site, randomized, double-blind, placebo-controlled clinical trial will be conducted in a university-based hospital in a laboratory setting and/or by telehealth. A total of 66 participants with chronic (>3 months) midportion or insertional AT will be randomized for the Tendinopathy Education of the Achilles (TEAch) study. All participants will complete progressive Achilles tendon loading exercises over 12 weeks and will be encouraged to continue with self-selected exercises as tolerated. All participants will complete 6-7 one-to-one sessions with a physical therapist to progress exercises in a standardized manner over 8 weeks. During the last 4 weeks of the intervention, participants will be encouraged to maintain their home exercise program. Participants will be randomized to 1 of 2 types of education (pain education or pathoanatomic), in addition to exercise. Pain education will focus on the biological and psychological mechanisms of pain within a biopsychosocial framing of AT. Pathoanatomic education will focus on biological processes within a more traditional biomedical framework of AT. Evaluation sessions will be completed at baseline and 8-week follow-up, and self-reported outcome measures will be completed at the 12-week follow-up. Both groups will complete progressive Achilles loading exercises in 4 phases throughout the 12 weeks and will be encouraged to continue with self-selected exercises as tolerated. Primary outcomes are movement-evoked pain during heel raises and self-reported function (patient-reported outcome measure information system—Physical Function). Secondary outcomes assess central nervous system nociceptive processing, psychological factors, motor function, and feasibility.

**Results:**

Institutional review board approval was obtained on April 15, 2019, and study funding began in July 2019. As of March 2020, we randomized 23 out of 66 participants. In September 2020, we screened 267 individuals, consented 68 participants, and randomized 51 participants. We anticipate completing the primary data analysis by March 2022.

**Conclusions:**

The TEAch study will evaluate the utility of pain education for those with AT and the effects of improved patient knowledge on pain, physical function, and clinical outcomes.

**International Registered Report Identifier (IRRID):**

DERR1-10.2196/19111

## Introduction

### Background

Achilles tendinopathy (AT) pain leads to decreased function and participation in work and recreation activities [1,2]. However, factors that contribute to the development and persistence of AT pain are not well understood. Recent evidence suggests that neurobiological pain processes in the peripheral and central nervous systems (CNS) contribute to chronic AT pain [3-7]. Factors that have been associated with AT include centrally mediated mechanisms such as elevated pain, psychological factors (fear of movement and pain catastrophizing) [5,8], and motor dysfunction (heel raise repetitions reduced by pain) [5]. However, this relationship is complex and bidirectional, as pain can also reduce function. In addition, there is mixed evidence for the presence of altered CNS regulation of nociceptive processing contributing to AT pain [4,5], with some studies indicating reduced conditioned pain modulation (CPM) and/or widespread decrease in pressure pain [6,7], whereas other studies indicate no difference compared with controls [4,5]. Peripheral mechanisms include nociceptive input, as evidenced by decreased pain pressure threshold (PPT) at the site of pain relative to multiple proximal and contralateral areas [4,5,7]. An improved understanding of how different physical therapy treatment approaches affect these mechanisms of AT pain could inform clinical practice.

### Objectives

Chronic musculoskeletal pain conditions, such as AT, can be associated with elevated levels of kinesiophobia and catastrophizing [9]. Given the frequent chronic duration of AT symptoms, fear avoidance and negative beliefs about movement and exercise may negatively impact patient compliance and outcomes of tendon loading exercises [10]. Pain education as part of physical therapy, with a biopsychosocial approach, has recently emerged as a promising component of care for many chronic musculoskeletal pain conditions. Greater patient understanding of their condition facilitates improved self-efficacy and management of symptoms while decreasing kinesiophobia and pain catastrophizing [9]. The standard of care for AT includes progress in tendon loading exercises, based on a high level of evidence [8,10-13]. In contrast, no clinical trials have evaluated the effects of pain education on pain and function in patients with Achilles tendon pain [14]. We hypothesize that a biopsychosocial approach to patient education, which addresses pain-related psychological factors and provides accurate information on adaptation, biology, and central pain mechanisms, will decrease movement-evoked pain and improve self-reported function at 8 weeks more than standard care for patients with AT, which is usually based on a pathoanatomical educational approach.

## Methods

### Overview

**Figure 1:**
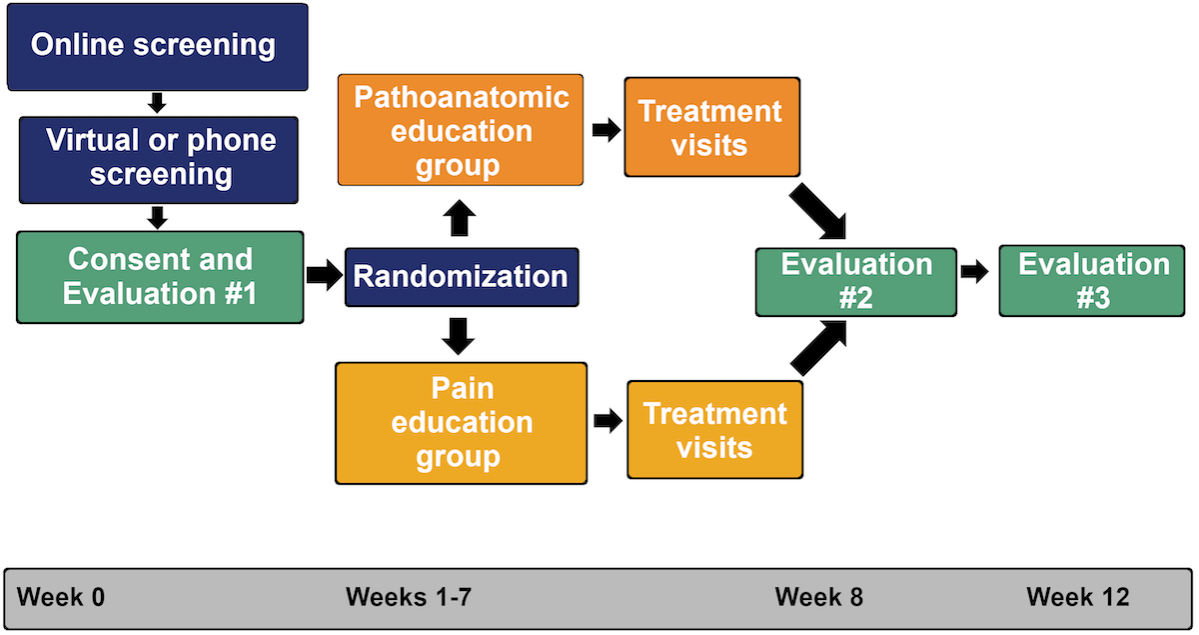
After consent and baseline measures at Evaluation #1, participants who satisfy all eligibility criteria are randomized to education group. A physical therapist provides education along with exercise over 6-7 individual treatment sessions. At 8-weeks participants complete Evaluation #2 to repeat all baseline measures. At 12-weeks the participants complete Evaluation #3, which consists of online surveys.

Tendinopathy Education on the Achilles (TEAch; NCT *04059146*) is a randomized, double-blind, placebo-controlled trial for individuals with chronic AT. The primary outcomes of movement-evoked pain and self-reported function will be represented as a change from baseline to 8-week follow-up (Figure 1). All participants will receive a progressive tendon loading exercise program and be assigned to either a pain education program or a pathoanatomical education program on AT pathology. The TEAch study has 2 primary aims and 2 exploratory aims. The first aim is to determine if an 8-week progressive tendon loading exercise program combined with pain education on AT is more effective at reducing movement-evoked pain and self-reported function than pathoanatomical education on AT. The second aim is to identify processes (altered CNS regulation of nociceptive input, changes in fear or pain beliefs, and improved motor function) that change over time with the intervention, regardless of education type. Our first exploratory aim is to identify if improvements in pain mechanism knowledge related to AT are associated with an improvement in pain and function. The second exploratory aim will identify if improvements in pain processes (altered CNS regulation of nociceptive input, changes in fear or pain beliefs, and improved motor function) are associated with an improvement in pain and function. In addition, a feasibility aim will gather information (recruitment, treatment fidelity, outcome capture rate, and adverse events [AEs]) to inform future clinical trials through both in-person and telehealth delivery of interventions on the outcomes of pain and function.

### Participants

Participants will be recruited through the University of Iowa and local community using mass emails, databases of participants previously enrolled in research studies, referrals from participants and collaborators in the Department of Orthopedics and Rehabilitation, and review of electronic medical records. All participants will be screened through a web-based survey and review of the medical records, if available, for inclusion and exclusion criteria (Textbox 1). Potential participants will be contacted via email and/or phone by the study coordinator. Participants will be consented at their first evaluation session, including a review of the purpose of the study, risks and benefits, procedures including confidentiality and interventions, and opportunity for participants’ questions. After informed consent, an experienced physical therapist will confirm a diagnosis of AT by clinical exam and ensure that all inclusion criteria are met (Textbox 1). Participants who do not meet the eligibility criteria at evaluation session 1 will be excluded from randomization to the treatment group. Participants will be compensated for completion of evaluation sessions and travel and parking for evaluation and treatment visits. The protocol was reviewed and approved by the Institutional Review Board (IRB) at the University of Iowa.

**Study eligibility criteria are assessed during web-based screening and phone or virtual screening. A clinical exam by a physical therapist is used to verify eligibility criteria at evaluation session 1.**

Inclusion criteria

- Primary source of pain localized to Achilles tendon insertion or midportion under load during clinical exam
- Localized pain 3/10 in the Achilles tendon (midportion, insertion, unilateral, or bilateral) during walking, heel raises, or hopping at evaluation session 1
- Pain that increases (>1 point on 11-point scale) with increasing load during evaluation session 1

Exclusion criteria

- Younger than 18 years of age
- Inability to read and write in English
- Achilles tendon pain for <3 months
- History of Achilles tendon rupture that was verified by surgical or conservative management
- History of invasive intervention (surgery and Tenex) for Achilles tendinopathy (AT) on more painful side
- Noninvasive treatment (physical therapy, nitroglycerine patch, iontophoresis, and injection) for AT in the past 3 months
- Diagnosed with systemic inflammatory conditions (eg, rheumatoid arthritis and ankylosing spondylitis), endocrine disorder with complications (eg, uncontrolled type 1 or 2 diabetes and diabetic peripheral neuropathy), and connective tissue disorder (eg, Marfan syndrome)
- Cardiovascular conditions that may be exacerbated by a 90-second submersion of hand in cold water (Raynaud’s and cold contact urticaria)
- History of taking fluoroquinolones in the past 3 months
- Foot and ankle pain primarily owing to other causes, such as posterior impingement, bursitis, paratendonitis, sural nerve injury, ankle osteoarthritis, and radicular or referred symptoms (pain, altered sensation, weakness, and altered reflexes), from lumbar spine into lower extremities
- Four step square test >15 seconds (in-person fall risk assessment)
- Cardiovascular condition that prevents participation in an exercise program

### Sample Size

The primary outcomes of this study were movement-evoked pain and self-reported function. The sample size is calculated to have sufficient statistical power for each primary outcome using a Bonferroni adjusted type I error rate of 0.025 (0.05/2 for 2 outcomes in aim 1). On the basis of findings by Moseley [15] for a randomized controlled trial (RCT) examining the effect of exercise and pain education for patients with chronic low back pain, we anticipate between-group differences with Cohen *d*≥0.36 for pain (between-group difference across 2 time points=0.75; 1.05 SD on the numeric pain rating scale; effect size of f=0.36; correlation between repeated measures=0.5) and self-reported function (between-group difference across 2 time points=1.95; 2.33 SD on low back pain–specific measure; effect size of f=0.42; correlation between repeated measures=0.5) [15]. Using these estimates, a sample size of 30 patients per group is needed to reach 80% power for the time-averaged difference between two group means in a repeated measures design with =.025 to detect a between-group effect size of 0.36. A final sample size of 60 is sufficient to detect estimated effect sizes for the outcome measures based on previously published differences or clinically meaningful minimal clinical difference/minimal clinically important difference and SD for the AT population (Table 1). We will consent 110 participants, estimating a 40% ineligibility rate after screening and 10% attrition rate between the first and second evaluation visits, to achieve a total of 66 participants consented and randomized.

**Table 1:** The primary outcome measures are movement-evoked pain during single limb heel raises and self-reported function on the patient-reported outcome measure information system computer adaptive testing for Physical Function 2.0. Secondary outcomes include conditioned pain modulation as an indicator of altered central nervous system nociceptive processing, Tampa Scale of Kinesiophobia as an indicator of pain-related psychology, and maximum number of single limb heel raises as an indicator of motor dysfunction.

Outcome			Published mean difference of MCD^a^ or MCID^b^ and SD		Estimated effect size
**Specific aim 1, powered to detect between-group effect sizes of** *d* **≥0.36, mean (SD)**					
	Movement-evoked pain (NPRS^c^) [16,17]	1.0 (1.9)		0.53	
	Function (PROMIS PF^d^) [18,19]	7.9 (9.0)		0.88	
**Specific** **aim 2, powered to detect within group effect sizes of** *d* **≥0.43, mean (SD)**					
	Altered CNS^e^ nociceptive processing (CPM^f^) [20]	84.0 (68.1)		1.23	
	Pain psychology (TSK^g^) [21]	5.6 (6.2)		0.90	
	Motor dysfunction (heel raises)	4.7 (10.0)		0.47	

^a^MCD: minimal clinical difference.

^b^MCID: minimal clinically important difference.

^c^NPRS: numeric pain rating scale.

^d^PROMIS PF: patient-reported outcome measurement information system—physical function.

^e^CNS: central nervous system.

^f^CPM: conditioned pain modulation.

^g^TSK: Tampa Scale of Kinesiophobia.

### Study Design

This is a randomized, double-blind, placebo-controlled trial with individuals who have chronic AT. Participants will be randomized to 1 of 2 groups: pain education or pathoanatomic education. All participants will receive the same progressive tendon loading exercise intervention. Each participant will complete 2 evaluation sessions where primary outcomes will be collected at baseline and 8-week follow-up, 6-7 treatment sessions with a physical therapist, and 1 evaluation session at 12-week follow-up with self-reported measures (Figure 1).

### Randomization

Participants will be randomized using a permuted block design with variable block sizes. Randomization will be stratified by sex and AT location (insertional and midportion). Randomization codes will be stored electronically and printed by a lab assistant and placed into opaque envelopes and sealed. Each envelope will be numbered in sequential order and stored separately from the recruiter, outcome assessor, and treating physical therapist. Immediately before each participant’s first treatment session, the treating physical therapist will either open an envelope or receive an email with the participant’s group allocation. Thus, randomization will occur after baseline assessment and remain concealed from participants for the duration of the study. Following all outcome collections, participants’ planned unmasking will be completed at 12 weeks, where participants will be informed of their group allocation by providing references and resources used for both education groups. Following completion of the study, participants will be offered the option of being sent a copy of the published findings, quarterly newsletters on study progress, and provided with any preliminary findings after study completion.

### Interventions

The intervention will occur over 12 weeks. The educational component will take place over the first 8 weeks during individualized treatment visits. The last 4 weeks will include maintenance of a home exercise program (HEP) with a phone call and email follow-up, if not reached by phone, from the physical therapist at 10 weeks to address any questions regarding exercise progression. The education and exercise treatments will be provided to both groups by the same unblinded physical therapist. Although a single treatment provider minimizes confounding social effects between physical therapists, an unblinded provider does allow for the potential of a bias in exercise progression between groups. The education programs, including videos, handouts, and review questions, are similar in length, style, and presentation of content, including the use of a script by a physical therapist to maximize consistency. All educational materials including handouts, weekly exercise records, and questionnaires will be provided through email and completed electronically by participants through an electronic data management system (REDCap). The education programs address participant knowledge on the causes of their pain as well as the overall importance of exercise to address tendon pathology. The main differentiating component will be based on the proposed mechanisms of pain. The pain education group will receive information that addresses concerns about fear of movement and pain catastrophizing, relates these psychosocial factors to their own experience with AT pain, and provides information on how tendon pathology is a potential (but not necessary) contributor to AT pain and that there is evidence that progressive loading is safe (Textbox 2). Key resources used to develop this pain education content include Explain Pain, Retrain Pain Foundation, and Cognitive Therapy for Chronic Pain [22-26]. The pathoanatomic education group will use a pathoanatomical model where Achilles tendon pathology is considered the primary contributor to pain (Textbox 3). Key resources used to develop this pathoanatomic education content include publicly available resources developed by the American Physical Therapy Association, the American Academy of Orthopaedics, and the FIFA Medical Network [14,27,28]. The fidelity of the intervention will be assessed by 2 steering committee members who will review a total of 10 recorded treatment visits and categorize them based on presumed participant group allocation and with a confidence rating of 0 (not confident at all) to 5 (completely confident) for the ability to determine participant group.

**Pain Educational Group treatment session themes and key messages related to Achilles tendinopathy. Homework assignments include an exercise log, multiple choice questions related to educational video content, and short-response questions to facilitate individualization of applying educational material.**

Progressive loading exercises for tendinopathy (same for both groups):

- Defining the term load for tendon pain rehabilitation
- Types of loads placed onto the Achilles tendon during various activities
- Use of symptoms 24 hour after completion of exercises to inform exercise dosage

Rethinking the role of exercise for Achilles tendinopathy (AT):

- Achilles tendon load capacity and role of exercise to increase capacity
- Progressive increase in Achilles tendon exercise intensity and duration
- Difference between AT and Achilles tendon rupture

Common tendon adaptations to loading:

- Common tissue adaptations seen on imaging including bone spurs, tendon calcification, and Haglund deformity
- Lack of correlation between pathology viewed on imaging and clinical presentation of pain/stiffness with AT

Factors influencing pain:

- Pain neurobiological processing including nociceptor activity and signal interpretation by the brain to produce sense of pain to protect from harm or danger
- Impact of psychological factors such as stress and context of whole pain experience

Understanding pain:

- Hypersensitivity of the peripheral and central nervous system and persistent pain
- The role of descending facilitation and inhibition on chronic pain conditions
- Recognition that persistent pain is multifactorial

Benefits of exercise for chronic musculoskeletal pain:

- Neurotransmitters and inflammatory mediates present with persistent pain
- Roles of exercise on improving immune system and neurotransmitter function to decrease pain
- Physical activity guidelines from the Department of Health and Human Services

**Pathoanatomic Education Group treatment session themes and key messages related to Achilles tendinopathy. Homework assignments include an exercise log, multiple choice questions related to educational video content, and short-response questions to facilitate individualization of applying educational material.**

Progressive loading exercises for tendinopathy (same for both groups):

- Defining the term *load* for tendon pain rehabilitation
- Types of loads placed onto the Achilles tendon during various activities
- Exercise progression and use of symptoms 24 hours after completion of exercises to inform exercise dosage

Effects of exercise on Achilles tendon pathology:

- Collagen tissue composition and common changes with tendinopathy
- Defining terminology of tendon pathology (tendinitis/tendinosis/tendinopathy)
- Role of exercise in addressing collagen tissue remodeling through progressive loading exercise

Soft-tissue and boney deformities associated with Achilles tendinopathy (AT):

- Prevalence and etiology of AT
- Presentation of radiographic images of common anatomical findings often associated with AT including Haglund deformity, bone spurs, and calcification within the Achilles tendon

Anatomical causes of AT pain:

- AT classification (midportion versus insertional)
- Continuum of mechanical tendon properties from healthy to tendon rupture
- Intrinsic and extrinsic factors which predispose tendon to dysfunction (age, activity level changes, foot mechanics, and repetitive trauma)

Understanding tendinopathy pathophysiology:

- Pathogenesis of AT
- Common imaging techniques used to identify pathology
- Components of clinical evaluation for AT diagnosis including patient history and physical examination

Whole body benefits of exercise:

- Impact of exercise on multiple systems throughout the body (immune system, cardiovascular system, and brain function)
- Physical activity guidelines from the Department of Health and Human Services
- Individualizing exercise goals

**Figure 2:**
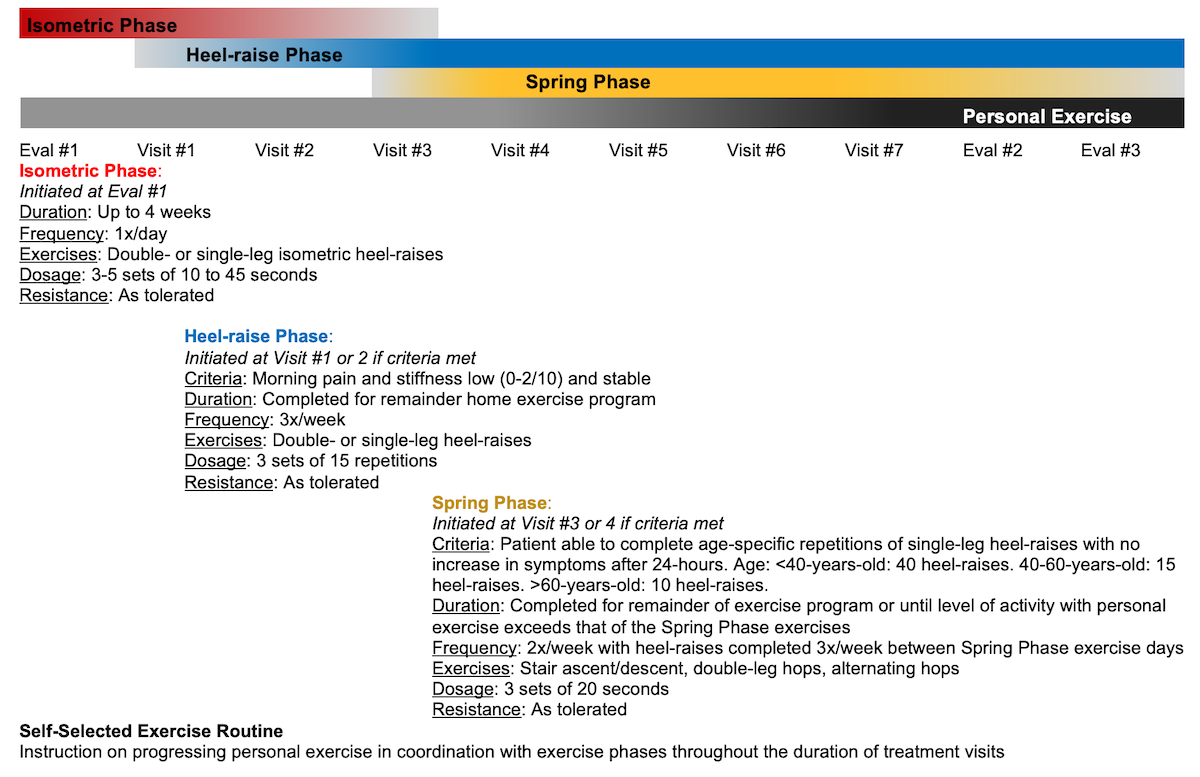
Progressive tendon-loading exercise program with 4 overlapping phases (isometric, heel raise, spring phase, and self-selected exercise). Participants are encouraged to maintain any ongoing personal exercise throughout the study, and then receive additional instruction on progression at final phase. Patient are instructed to monitor for symptom increase within 24-hour window after completion of exercises.

Each participant will receive the same standardized therapeutic exercise program where progression will be individualized to each participant based on pain levels, the physical therapist’s clinical judgment, and predetermined physical function criteria (Multimedia Appendix 1). The exercise intervention is based on evidence supporting the use of isometric exercise as a safe starting point for tendon loading and for pain relief as well as progressive isotonic tendon loading and restoring the spring-like function of Achilles [14,29,30]. The exercise program will consist of 4 phases with a progressive increase in tendon loading beginning with isometrics, progressing to concentric/eccentric heel raises, a functional spring phase, and a self-selected exercise routine (Figure 2). Throughout study participation, we will monitor the pain level using the 11-point verbal numeric pain rating scale. If a participant has 4/10 pain, then we will offer them to take a break or modify the activity. Blood pressure will be assessed before and after initiating aerobic activity during the spring phase with in-person visits. Participants will not be eligible for telehealth visits if they report symptoms indicating the need for in-person blood pressure monitoring, including (1) inconsistent use of hypertension medications and/or (2) any recent/current associated symptoms with uncontrolled hypertension. Exercise will be stopped according to the American College of Sports Medicine guidelines [31]. Participants will be asked to refrain from other invasive and noninvasive interventions during enrollment, including surgery, injection at the Achilles, and other forms of rehabilitation. Participants will be offered the option of follow-up visits via a telehealth format. This option will be provided to participants who are unable to attend owing to illness, limited transportation, or any other circumstance that may restrict the participants’ ability to complete in-person visits.

### Outcomes

Outcomes are described by the specific aim and timeframe collected in Tables 2 and 3. Additional outcomes collected for exploratory aims and examining the feasibility of future clinical trials are provided in Table 4. At evaluation session 1 (0 weeks) and evaluation session 2 (8 weeks), participants will complete functional testing in the following order: walking at a self-selected pace, walking at a standardized pace (Froude 4) [32], heel raises, and hops. Participants will then complete the Tampa Scale of Kinesiophobia (TSK) and Pain Catastrophizing Scale with instruction to think about any pain or discomfort in their Achilles tendon during walking, heel raises, and hopping. Participants who complete in-person evaluation sessions will conclude the session with quantitative sensory testing.

**Table 2:** For specific aim 1, study outcomes will be assessed pretreatment (week 0 at evaluation session 1), after completion of education combined with exercise (week 8 at evaluation session 2), and after 4 weeks of continuing the home exercise program at home (week 12 at evaluation session 3).

Outcome			Description	Time (weeks)		
				0	8	12
**Specific aim 1**						
	**Pain**					
		Movement-evoked pain during heel raises^a^	Participants will rate the intensity and location of their Achilles tendon pain using the 11-point NPRS^b^ during maximum number of single limb heel raises and 3 single limb hops [33,34]. Pain will be assessed during movement activities using the NPRS which consists of a 0-10 scale where 0 represent “no pain” and 10 represents “worst pain imaginable.” Test-retest reliability: *r=*0.67-0.96. Convergent validity: *r=*0.79-0.95 [34]	X^c^	X	—^d^
		Anticipated movement-evoked pain	Participants will rate the *anticipated* intensity of their Achilles tendon pain using a 101-point NPRS before heel raises and before hopping [33]	X	X	X
		Tendon stiffness	Participants will rate the average duration of stiffness in the Achilles tendon in the morning from 0 to ≥100 min over the past week	X	X	X
		Global rating of change	Anticipated and final change in the overall condition of the Achilles tendon on a 15-point scale. Test-retest reliability: ICC^e^ 0.90. Face validity: *r=*0.72-0.90 [35]	X	X	X
		Anticipated change in symptoms	Anticipated and final change in symptoms on a 15-point scale following physical therapy [35]	X	X	X
	**Function**					
		Global physical function^a^	Self-reported physical function will be assessed with the patient-reported outcomes measurement information system 2.0 and computer adaptive test physical function, which has been used in orthopedic and Achilles tendon populations [18,19]. Internal consistency reliability: *r*=0.96. Convergent validity: r=0.68-0.79 [36]	X	X	X
		Achilles tendon function	Function will be assessed with the Victorian Institute of Sport Assessment-Achilles questionnaire as a symptom severity measure with activity that is specific to patients with AT^f^. Test-retest reliability: *r*=0.93. Construct validity: *r*=0.58 [37]	X	X	X
		Activity log	Self-reported activity, including type (aerobic and strengthening) and duration per week on the home exercise log for first week compared with last week of exercise-education program	—	X	—
		Activity level	International Physical Activity Questionnaire Short-Form and self-reported activity levels including number of days and time spend completing vigorous or moderate activity, walking or sitting over past 7 days. Test-retest reliability: *r*=0.32-0.88. Criterion validity: *r*=0.12-0.57 [38]	X	X	X
		Patient-specific functional scale	Patient-reported ability to complete self-selected activities on an 11-point scale: 0=unable to perform activity and 10=able to perform activity at previous level. Test-retest: *r*=0.84 [39]. Concurrent validity: *r*=0.55-0.83 [40]	X	—	X

^a^Primary outcomes.

^b^NPRS: numeric pain rating scale.

^c^Outcome collected at timepoint.

^d^Outcome not collected at timepoint.

^e^ICC: intraclass correlation coefficient.

^f^AT: Achilles tendinopathy.

**Table 3:** For specific aim 2, study outcomes will be assessed pretreatment (week 0 at evaluation session 1), after completion of education combined with exercise (week 8 at evaluation session 2), and after 4 weeks of continuing the home exercise program at home (week 12 at evaluation session 3).

Outcome			Description	Time (weeks)		
				0	8	12
**Specific Aim 2**						
	**Altered CNS^a^** **nociceptive processing**					
		Conditioned pain modulation at site of Achilles tendon pain CPM^b^ response on contralateral side at the hamstring Widespread pain indicated by the Pain Pressure Threshold	PPTs^c^ will be collected bilaterally at the Achilles (centered around the most painful region) and hamstring with a pressure algometer (Somedic Algometer Type II, Horby Sweden, probe 1 cm^2^) at a rate of 50 kPa per sec. PPTs will be collected at the Achilles (painful side) and Hamstring (contralateral side) during the conditioning stimulus. The algometer will be positioned perpendicular to the skin with force applied in a posterior to anterior direction. The PPT value will be the average of a series of 3 repeated trails per site. The site for the Achilles on the painful side will be at the location reported to be most painful and the contralateral side will be at a similar distance from the tendon insertion on the contralateral side. The PPT for the hamstring will be on the semitendinosus/semimembranosus tendon located 3 cm from the crease along the back of the knee (test-retest reliability: ICC^d^ 0.93-0.95) [5]. To minimize temporal summation, the interstimulus interval will be ≥10 seconds. PPTs are collected with hand in room temperature water and during the conditioning stimulus starting at 20 seconds. The allocation of the Achilles and hamstring as site 1 versus site 2 will be randomized as well as order of collecting PPT during room temperature water versus during the conditioning stimulus. Participants are instructed to press a trigger first when the pressure becomes painful (pain >0/10). For CPM testing, the participant’s right hand is immersed up to the wrist in ice water (6 °C [SD 0.5]) for a total of 2 min as a conditioning stimulus. The intensity of the conditioning stimulus is maintained by visually monitoring temperature (brand of thermometer) throughout CPM testing and circulating the water with an aquarium air pump. Participants will also rate the pain in their hand at 5 seconds, 20 seconds, and 120 seconds (test-retest reliability: ICC 0.86-0.93) [5]	X^e^	X	—^f^
		Widespread pain indicated by Body Map	Participants will be asked to select the number of areas where they have experienced persistent or recurrent pain in the past 3 months using the Michigan Body Map [41]	X	X	—
	**Psychological factors**					
		Fear of movement	Participants will be asked to complete the TSK^g^ immediately following walking, heel raises, and hops completed during the evaluation and rate current level of fear about movement causing pain and injury during these activities. Test-retest reliability: *r*=0.64-0.89. Validity: *r*=0.70-0.81 [42,43]. Scored 17-68, a score of 37 indicates clinically meaningful levels of kinesiophobia [44]	X	X	—
		Pain catastrophizing	The pain catastrophizing scale (PCS) rates on a 5-point scale how often a participant has catastrophizing thoughts toward pain. Test-retest reliability: *r*=0.87. Validity: *r*=0.56 [45-48]. The PCS consists of 13 items and is scored 0 to 52 with a score >30 reported to demonstrate high catastrophizing [45]	X	X	—
		Self-efficacy Anxiety Depression	The PROMIS CAT^h^ 1.0 to assess for pain management self-efficacy, anxiety, and depression [21,42,45,49-51]. Self-efficacy: Validity: *r*=0.56-0.75 [50]. Anxiety: Test-retest reliability: *r*=0.822. Validity: *r*=0.41 [51]. Depression: Test-retest reliability: *r*=0.859. Validity: *r*=0.41 [51]	X	X	—
	**Motor function**					
		Single limb heel raises	We will use a 10-segment kinematic model of the body to quantify 3D motion. Participants will perform tasks over a force plate, flush with the floor, which provides 3D ground reaction forces. Plantar flexor endurance will be quantified with the maximum number of repetitions as well as the repeated heel raise work test [52], calculated using heel height (measured with a calcaneal marker) and force (measured with a force plate; test-retest reliability: ICC 0.83) [5]	X	X	—
		Counter movement jump	The vertical jump test will be used to quantify maximum jump height and peak ankle power [53]. Participants will be instructed to place their hands on their hips, bend their knee, and jump as high as possible on one leg. They will also try to take off and land in the same place (test-retest reliability: ICC 0.97) [5]	X	X	—
		Walking	Participants will walk at a self-selected (as if at home or work) and at a standardized speed (Froude 4) to capture use of the plantar flexors (peak ankle power) with this low-level daily activity. For in-person sessions, a minimum of 3 representative trials are collected per side for each gait speed. For virtual evaluation sessions, participants are asked to walk for 5 min in their home	X	X	—

^a^CNS: central nervous system.

^b^CPM: conditioned pain modulation.

^c^PPT: pain pressure threshold.

^d^ICC: interclass correlation coefficient.

^e^Outcome collected at timepoint.

^f^Outcome not collected at timepoint.

^g^TSK: Tampa Scale of Kinesiophobia.

^h^PROMIS CAT: patient-reported outcome measure information system computer adaptive test.

**Table 4:** For exploratory aim 1, the primary outcomes include primary outcomes for specific aim 1 and treatment fidelity. For exploratory aim 2, the primary outcomes include primary outcomes for specific aim 1 and specific aim 2.

Title and description					Time collected						
					Pre		During		Week 8		Week 12
**Feasibility aim**											
	**Recruitment**										
		**Rate of recruitment (participants enrolled) per year**		X^a^		X		X		X	
			Number of participants screened per month	X		X		X		X	
			Number of participants enrolled per month	X		X		X		X	
			Number of participants lost to follow-up per month	X		X		X		X	
			Rate of retention per year	X		X		X		X	
	**Treatment fidelity**										
		**Adequate knowledge of education program at 8-week follow-up**		X		—^b^		X		—	
			Rating of audio recording by external reviewers for confidence of participant group allocation on 0-5 scale	—		X		—		—	
			Time participants spent in treatment sessions	—		X		—		—	
			Time participants spent doing education homework between sessions	—		—		X		—	
			Adequate blinding of participants to bias of research team “At the beginning of the study, you were randomized to receive either Education A or Education B. We believe Education A is more helpful for recovery from Achilles tendinopathy than Education B. Which Education do you think that you received?” (A, B, I don’t know)	—		—		X		—	
			Duration of each exercise phase	—		X		—		—	
			Highest loading level attained at each exercise phase	—		X		—		—	
			Therapeutic alliance “What I was doing in physical therapy gave me new ways of looking at my problem.” “I was confident my physical therapist’s ability to help me.” “My PA and I were working towards mutually agreed upon goals.” (7-point scale from Never to Always)	—		—		X		—	
			Adherence to exercise program			X					
	**Outcome capture rate**										
		**Percentage of missing data per outcome**		X		X		X		X	
			Reasons for missing data	X		X		X		X	
	**AEs^c^**										
		Frequency and type of AEs		X		X		X		X	
**Other prespecified outcomes**											
	**Demographics**										
		Date of birth, sex, race/ethnicity, height/weight/BMI, description of AT^d^ symptoms, goals for physical therapy, previous experience with conservative care, comorbidities		X		—		—		—	
	**Four square step test**										
		Participants will perform a series of steps in a square formation. The duration of time needed to complete the step reflects dynamic balance and mobility		X		—		—		—	
	**Ultrasound imaging of tendon pathology**										
		Ultrasound imaging will be used to quantify tendon thickness, echogenicity, presence of osteophytes/bone spur		X		—		X		—	
	**Medication**										
		History, current use, and dose of all routine medications and specific questions about opioid use		X		—		X		X	
	**Treatment history**										
		Treatments previously tried and if they were effective at reducing pain		X		—		—		—	
	**Mode of participants’ complete visits**										
		Percentage of participants who completed all visits (evaluation sessions 1 and 2 and follow-up visits) through in-person visits compared with those who completed a percentage of visits through telehealth format				—		—		X	

^a^Outcome collected at timepoint.

^b^Outcome not collected at timepoint.

^c^AEs: adverse events.

^d^AT: Achilles tendinopathy.

### Statistical Analysis

We will use a modified intention-to-treat analysis to examine the treatment effect for all outcome measures on participants based on group randomization. Characteristic comparisons will also be completed for those patients who remained in the study versus those who dropped out to determine if data at subsequent time points were consistent with missing at random data. For aim 1, we will compare differences between education groups for changes in movement-evoked pain and self-reported function from baseline to 8 weeks (primary endpoint) and 12 weeks using a linear mixed model for repeated measures. For aim 2, we will use a linear mixed model for repeated measures for intervention effect within groups on central pain mechanisms from baseline to 8 weeks. Secondary outcomes for pain, function, altered CNS nociceptive processing, psychological factors, motor control, pain, and function will be analyzed with a linear mixed model for repeated measures. Demographics, ultrasound imaging, and treatment history will be used to describe the sample and will be covariates in the analysis if different between groups. Secondary analyses of sex will examine for potential sex-based differences to inform sample size estimates for future clinical trials. Another secondary analysis of AT type (midportion vs insertional) will also be assessed to inform recruitment strategies for future clinical trials.

Exploratory aims 1 and 2 will examine whether changes in participant knowledge of pain education and central pain mechanisms, including altered CNS regulation of nociceptive input, changes in fear or pain beliefs, and improved motor function are associated with pain and function. For exploratory aim 1, we will use Pearson correlations between changes in knowledge from baseline to final evaluation (percentage of correct responses to pain education multiple choice questions) and changes in pain and function separately. For exploratory aim 2, we will examine Pearson correlations between changes in central pain mechanisms and changes in pain and function separately.

To evaluate the feasibility of future clinical trials, we will use descriptive statistics to assess patient retention, participant recruitment numbers, treatment fidelity, and patient adherence to their exercise program. To evaluate for potential confounders of treatment effect between groups, we will assess differences in duration of treatment sessions, time spent completing their HEP, time participants spent in each phase of their exercise program, percentage of participants who believed they were receiving the education program that the research team believed to be more effective, and the highest level reached within each exercise phase (half body weight, body weight, and machine weight) using two independent samples *t* tests, Wilcoxon rank-sum test for continuous variables, or Chi-square or Fisher exact test for categorical variables, as appropriate.

### Alterations to Study After Initiation

From March 17 to July 15, 2020, in-person human subjects research was suspended in accordance with the University of Iowa policy related to COVID-19. We conferred with the Data Safety Monitoring Board (DSMB) and the safety officer on protocol changes to continue the clinical trial via telehealth. These protocol changes were approved by the IRB at University of Iowa on March 17, 2020. The intervention content and the primary outcomes of movement-evoked pain and self-reported function were not altered, yet the transition to a virtual format affected screening, evaluation, and mode of delivering treatment as outlined below. Participants who did not pass the additional virtual screening questions were categorized as *delayed owing to COVID* and rescreened once in-person human subjects research resumed.

#### Virtual Screening

Modifications to the screening process were included at initiation of intervention delivery via telehealth due to COVID-19 to ensure participant safety during completion of the exercise program:

- Fall risk was completed using the Stopping Elderly Accidents, Deaths, and Injuries (STEADI; score>4) [54] rather than in-person using the 4-square step test.
- Symptoms indicating the need for in-person blood pressure monitoring: (1) inconsistent use of hypertension medications and/or (2) any recent/current associated symptoms with uncontrolled hypertension.
- Unable to successfully complete virtual visits with a webcam and/or prefer only in-person visits.

#### Evaluation

Additional data collected via Zoom was completed to continue primary outcome collection during the telehealth format. The telehealth format did not permit imaging or QST data collection:

- Two-dimensional kinematics were collected via Zoom instead of 3D kinematics and kinetics.
- Ultrasound imaging and quantitative sensory testing were not completed.

#### Treatment

All treatment visits were completed on the web. There were no changes in the educational or exercise program content. The highest level of isometric and heel raise phases of the exercise program requires the use of an externally applied load via a Smith machine or weighted backpacks. Participants without access to a Smith machine in a gym, owing to lack of membership or COVID-19, were offered weighed backpacks.

Given that the educational materials (videos, handouts, and logs) had always been provided to participants via REDCap, the initial design of the intervention facilitated the transition of individualized discussion of materials from in-person to a virtual format. Since July 15, 2020, the option to complete virtual treatment visits remains available to participants who are unable to attend owing to illness, limited transportation, or any other circumstance that may restrict the participants’ ability to complete in-person visits.

In addition, the pandemic has had negative effects on mental and physical health. Among adults in the United States, from June 24 to 30, 2020, 31% reported symptoms of anxiety disorder or depressive disorder [55]. A study including 906 health care workers at 5 major hospitals within the period of February 19 to April 20, 2020, reported that depression and anxiety were associated with the presence of physical symptoms, including musculoskeletal pain [56]. As a biopsychosocial approach addresses the interaction between mental and physical health, the pandemic may magnify the differential effects of pain education combined with exercise compared with a pathoanatomic education.

The psychosocial effects of this pandemic and study protocol changes motivated 2 additional exploratory analyses to (1) examine potential confounding effects of a pandemic and virtual participation on outcomes and (2) determine the feasibility of a clinical trial via telehealth. Our study sample can be divided into 3 groups of participants: all in-person visits (September 21, 2019, to March 16, 2020), all visits via telehealth (March 17 to July 15, 2020), and a mix of visits in-person and via telehealth during the pandemic (July 16 to study completion). To evaluate the potential interactions of the pandemic and virtual participation on the intervention, the changes in the primary outcomes (pain and disability) and psychological outcomes (fear of movement, pain catastrophizing, self-efficacy, depression, and anxiety) will be compared between the 3 pandemic groups within each treatment arm (pathoanatomic vs pain education). To examine the feasibility of future clinical trials using a virtual format, recruitment rates, retention, adherence to HEP, and AEs will be compared between the 3 pandemic groups (all in-person, all virtual, and mixed).

### Ethics

All patient data will be stored in electronic records kept on a network drive of the Department of Physical Therapy and Rehabilitation Science at the University of Iowa and on REDCap. Access will be restricted to only the research team who will comply with the confidentiality of patient information consistent with the Health Insurance Portability and Accountability Act guidelines. An independent safety officer will review all AEs, serious AEs, unanticipated problems, and any protocol deviations affecting safety on a quarterly basis. The DSMB will meet yearly with the study team. The DSMB will convene annually to review the data, recruitment, and safety of subjects. This review will include a discussion of the allocation concealment process to ensure that concealment is done at the last minute and review of records to ensure that the proper random sequence was used. The review will also include reports of AEs, serious AEs, protocol deviations or violations, and unanticipated problems. Recruitment and retention will also be reviewed.

### Role of Funding Source

Funding for this study was provided by the National Institute of Arthritis Musculoskeletal and Skin Disease research grant R00 AR071517 and the Collaborative Research Grant from the International Association for the Study of Pain (IASP). Research reported in this publication was supported by the National Center for Advancing Translational Sciences of the National Institutes of Health under Award Number UL1TR002537. These funding sources had no role in the study design, collection, analysis/interpretation of data, or decision on submission for publication. The content is solely the responsibility of the authors and does not necessarily represent the official views of the National Institutes of Health.

## Results

### Overview

Institutional review board approval was obtained on March 15, 2019, and study funding began on July 1, 2019. The TEAch study began enrollment on September 17, 2019. As of March 2020, we randomized 23 out of 66 participants. In September 2020, we screened 267 individuals, consented 68 participants, and randomized 51 participants. We anticipate to complete the primary data analysis by March 2022 and will submit the results for primary outcomes no later than 1 year after the primary completion date on ClinicalTrials.gov (NCT: 04059146) and Open Science Framework (JF2XU).

### Individual Participant Data Sharing Plan

In compliance with FAIR (findability, accessibility, interoperability, and reusability) data principles, data will be deposited at the University of Iowa open-access institutional repository, Iowa Research Online. The repository is open access and maintained by the Libraries at the University of Iowa for the preservation and sharing of intellectual work of faculty, students, and staff. The IPD will be available to other researchers for the primary outcomes. Data sets will be accompanied with appropriate descriptive, technical, and administrative metadata to facilitate discovery and scholarly reuse, and will be assigned unique digital object identifiers (DOIs) that can be incorporated into publications and cited in the literature. Metadata will be included in the data records in the repository through readme files and structured information following the DataCite metadata schema.

## Discussion

Improved understanding of pain mechanisms and reconceptualization of pain as protective through patient education is recommended for individuals with chronic musculoskeletal conditions [57-59]. Although psychological factors have been explored in patients with AT [5,8,60], no study has examined the effect of patient education on these factors in patients with chronic AT. Previous reviews indicate that pain education alone is not sufficient to reduce pain and disability by a clinically meaningful amount [9,59]. However, a recent meta-analysis indicated that pain education combined with exercise and provided over a longer time frame had a larger effect on pain and disability [61]. This clinical trial will compare the effect of pain education combined with exercise over an 8-week period on movement-evoked pain and self-reported function in patients with AT with pathoanatomical education. Moreover, this RCT will examine changes in altered CNS regulation of nociceptive input, changes in fear or pain beliefs, and improved motor function before and after physical therapy to determine how these pain mechanisms are affected by care. We aim to advance care for this patient population through improved understanding of how education combined with exercise affects clinical outcomes for patients with AT.

## Appendix

Multimedia Appendix 1 Exercise progression criteria.

Multimedia Appendix 2 Peer-review report by AMS.

## Abbreviations

AE: adverse event
AT: Achilles tendinopathy
CAT: computer adaptive test
CNS: central nervous system
CPM: conditioned pain modulation
DSMB: Data Safety Monitoring Board
HEP: home exercise program
IASP: International Association for the Study of Pain
ICC: interclass correlation coefficient
NIH: National Institutes of Health
PPT: pain pressure threshold
PROMIS: patient-reported outcome measure information system
TEAch: Tendinopathy Education of the Achilles
