# Supplementary material for: Effect of Pain Education and Exercise on Pain and Function in Chronic Achilles Tendinopathy: Protocol for a Double-Blind, Placebo-Controlled Randomized Trial
Source: JMIR Res Protoc. 2020 Nov 3;9(11):e19111. doi: 10.2196/19111 (PMC7678911; doi:10.2196/19111)
Supplement: Multimedia Appendix 1 [file resprot_v9i11e19111_app1.pdf]

TEAch Exercise Progression

Isometric Phase – Weeks 1-2 including Evaluation #1 and Treatment Sessions 1-2 (no more than 4 weeks)

- Frequency of HEP: Everyday

Patient goal: Completion of 5 sets of 45 second isometric holds. 1 minute rest between sets. 7x/week

- Duration: Minimum of 1 week. Maximum of 4 weeks.
- Evaluation #1:
  - Initiation of isometric exercise

    Question 1- Can patient complete 15 seconds of double-leg isometrics comfortably with good form and no worsening of symptoms?

      No: Rx: Seated isometrics for up to 5 sets of 45 seconds

      Yes: Question 2- Can patient complete 15s of single-leg isometric exercise comfortably?

    - No: Rx: Double-leg isometric exercises for up to 5 sets of 45 seconds
    - Yes: Question 3- Can patient complete 5 sets of 45s of single-leg isometric?
      - No: Rx: Single-leg isometric for up to 5 sets of 45 seconds
      - Yes: Rx: Weighted isometrics
        - Note for all "weighted" exercises. Ideally done with weight equally distributed anterior and posterior to center of mass. Can use Smith machine at gym or 2 backpacks (one anterior and one posterior)

- Treatment Session #1:
  - Frequency of HEP: 7x/week
  - Isometric Progression:

    Question 1- Can patient complete 15 seconds of double-leg isometrics comfortably with good form and no worsening of symptoms?

      No: Rx: Seated isometrics for up to 5 sets of 45 seconds

      Yes: Question 2- Can patient complete 15s of single-leg isometric exercise comfortably?

    - No: Rx: Double-leg isometric exercises for up to 5 sets of 45 seconds
      - Note: If patient on double-leg isometrics for more than one week then can add weight-shifts as a mini-progression before single-leg
    - Yes: Question 3- Can patient complete 5 sets of 45s of single-leg isometric?
      - No: Rx: Single-leg isometric for up to 5 sets of 45 seconds
      - Yes: Rx: Progress to heel raises early (see initiation at Tx_2 below)

- Treatment Session #2: Initiation of heel-raises
  - Frequency of HEP: Isometrics: 4x/week. Heel-raises: 3x/week
  - Isometric Progression:

    Question 1- Can patient complete 15 seconds of double-leg isometrics comfortably?

      No: Rx: Seated isometrics for up to 5 sets of 45 seconds

      Yes: Question 2- Can patient complete 15s of single-leg isometric exercise comfortably?

    - No: Rx: Double-leg isometric exercises for up to 5 sets of 45 seconds
    - Yes: Question 3- Can patient complete 5 sets of 45s of single-leg isometric?
      - No: Rx: Single-leg isometric for up to 5 sets of 45 seconds
      - Yes: Rx: Progress to heel raises early (see initiation below)

- Initiation of heel-raises:
    1. Criteria for progression: Patient must report morning pain and stiffness as either low (0-2/10) or stable.
    2. Duration of Heel-raise phase: Completed through remainder of course of PT sessions
    3. Initiation of heel-raise phase:
       Question 1- Can patient complete 5 double-leg heel-raises comfortably?
  - No: Rx: Seated heel-raises for up to 3 sets of 15 as tolerated
  - Yes: Question 2- Can patient complete 5 single-leg heel-raises comfortably?
    - No: Rx: Double-leg heel-raises up to 3 sets of 15
    - Yes: Question 3- Can patient complete 3 sets of 15 single-leg heel-raises comfortably, slowly, and to full height?
      - No: Rx: Single-leg heel-raises up to 3 sets of 15
      - Yes: Rx: Weighted single-leg heel-raises up to 3 sets of 15

Heel-raise Phase: Weeks 1-4 including Treatment Sessions 1-4

- Criteria: Patient must report morning pain and stiffness as either low (0-2/10) or stable.
- Frequency of HEP: 3x/week
- Duration: Completed through remainder of course of PT sessions
- Patient goal: 3 sets of 15 repetitions, 1 minute rest between sets, 3x/week

- Treatment Session 3:
  - Frequency of HEP: 3x/week
  - Heel-raise progression:
    Question 1- Can patient complete 5 double-leg heel-raises comfortably?
  - No: Rx: Seated heel-raises for up to 3 sets of 15 as tolerated
  - Yes: Question 2- Can patient complete 5 single-leg heel-raises comfortably?
    - No: Rx: Double-leg heel-raises up to 3 sets of 15
    - Yes: Question 3- Can patient complete 3 sets of 15 single-leg heel-raises comfortably?
      - No: Rx: Single-leg heel-raises up to 3 sets of 15
      - Yes: Rx: Weighted HR & Progress to Spring Phase early

- Treatment Session 4: Initiation of Spring-phase
  - Frequency of HEP: Heel-raises: 3x/week, Spring-phase: 2x/week with 1 day rest between
  - Heel-raise progression:
    Question 1- Can patient complete 5 double-leg heel-raises comfortably?
  - No: Rx: Seated heel-raises for up to 3 sets of 15 as tolerated
  - Yes: Question 2- Can patient complete 5 single-leg heel-raises comfortably?
    - No: Rx: Double-leg heel-raises up to 3 sets of 15
    - Yes: Question 3- Can patient complete 3 sets of 15 single-leg heel-raises comfortably?
      - No: Rx: Single-leg heel-raises up to 3 sets of 15
      - Yes: Rx: Weighted HR & Progress to Spring Phase early
  - Transition from heel-raises to spring-phase
    1. Criteria for progression: Ability to complete single-leg heel-raises with no increase in symptoms the next day. Number of heel-raises dependent upon age. <40-years-old: 25 heel-raises. 40-60-years-old: 15 heel-raises. >60-years-old: 10 heel-raises. (Jan 2005)
    2. Initiation of Spring-Phase:

- Question 1- Can the patient comfortably complete 5 flights of slow stairs with heels up?
    - No: Slow stairs as tolerated with 1 minute rest between sets
    - Yes: Rx: Slow stairs up to 10 flights with 1 minute rest between sets and instruct in double leg hops with 3 sets of 5 seconds

<u>Spring Phase</u>: Weeks 3-8 including Treatment Sessions 3-6

- Criteria: Ability to complete single-leg heel-raises with no increase in symptoms the next day. Number of heel-raises dependent upon age. <40-years-old: 25 heel-raises. 40-60-years-old: 15 heel-raises. >60-years-old: 10 heel-raises. (Jan 2005)
- Frequency: 2x/week. Heel-raises completed 3x/week between Spring-Phase exercise days
- Patient Goals for specific intervention:
    - Slow stairs: 10 flights of stairs
    - Slow double leg hops: 3 set of 20 seconds
    - Fast stairs: 6 flights of stairs
    - Fast double leg hops: 3 sets of 20 seconds
    - Alternating leg hops: 3 sets of 20 seconds

- <u>Treatment Session 5:</u>
    - Frequency: 2x/week. Heel-raises completed 3x/week between Spring-Phase exercise days
    - Spring-Phase Progression:
        - Question 1- Can the patient comfortably complete 10 flights of slow stairs?
            - No: Slow stairs as tolerated with 1 minute rest between sets
            - Yes: Rx: Progress to double leg hops with 3 sets of 5-20 seconds

        - Question 2- Can the patient complete 3 sets of 20s slow double leg hops (non-continuous)?
            - No: Slow double leg hops with 3 sets of 5-20 seconds with 1 min rest between sets
            - Yes: Rx: Progress to up to 3 flights of fast stairs with heels up

<u>Treatment Session 6:</u>

- Frequency: 2x/week. Heel-raises completed 3x/week between Spring-Phase exercise days
- Spring-Phase Progression: (*assume all have had at least 1 week of slow stairs by this point*)
    - Question 1- Can the patient complete 3 sets of 20s slow double leg hops (non-continuous)?
        - No: Slow double leg hops with 3 sets of 5-20 seconds with 1 min rest between sets
        - Yes: Rx: Progress to up to 6 flights of fast stairs

    - Question 2- Can the patient complete 6 flights of fast stairs?
        - No: Fast stairs as tolerated with 1 minute rest between sets
        - Yes: Progress to 3 sets of 5 second fast, continuous double leg hops

    - Question 3- Can patient do 3 sets of 20s fast double leg hops?
        - No: Fast double leg hops, 3 sets of 5-20 secs
        - Yes: Rx: Alternating leg hops up to 3 sets of 20 seconds with 1 minute between sets
