# Supplementary material for: Effect of Pain Education and Exercise on Pain and Function in Chronic Achilles Tendinopathy: Protocol for a Double-Blind, Placebo-Controlled Randomized Trial
Source: JMIR Res Protoc. 2020 Nov 3;9(11):e19111. doi: 10.2196/19111 (PMC7678911; doi:10.2196/19111)
Supplement: Multimedia Appendix 2 [file resprot_v9i11e19111_app2.pdf]

SUMMARY STATEMENT

(Privileged Communication)

PROGRAM CONTACT:
Charles Washabaugh
301-496-9568
washabac@mail.nih.gov

Release Date: 12/02/2016
Revised Date:

Application Number: 1 K99 AR071517-01

Principal Investigator

CHIMENTI, RUTH LOUISE PORTER

Applicant Organization: UNIVERSITY OF IOWA

Review Group: AMS
Arthritis and Musculoskeletal and Skin Diseases Special Grants Review Committee

Meeting Date: 11/03/2016
Council: JAN 2017
Requested Start: 04/03/2017

RFA/PA: PA16-193
PCC: 3 B

Dual IC(s): HD

Project Title: Mechanism-based treatment approach to chronic tendinopathy pain

SRG Action: Impact Score:20
Next Steps: Visit http://grants.nih.gov/grants/next_steps.htm
Human Subjects: 30-Human subjects involved - Certified, no SRG concerns
Animal Subjects: 10-No live vertebrate animals involved for competing appl.
Gender: 1A-Both genders, scientifically acceptable
Minority: 1A-Minorities and non-minorities, scientifically acceptable
Children: 3A-No children included, scientifically acceptable
Clinical Research - not NIH-defined Phase III Trial

Project Year	Direct Costs Requested	Estimated Total Cost
1	79,804	80,914
2	80,475	81,594
3	249,000	252,462
4	249,000	252,462
5	249,000	252,462
TOTAL	907,279	919,893

ADMINISTRATIVE BUDGET NOTE: The budget shown is the requested budget and has not been adjusted to reflect any recommendations made by reviewers. If an award is planned, the costs will be calculated by Institute grants management staff based on the recommendations outlined below in the COMMITTEE BUDGET RECOMMENDATIONS section.

1K99AR071517-01 Chimenti, Ruth

SCIENTIFIC REVIEW OFFICER'S NOTE

RESUME AND SUMMARY OF DISCUSSION: This is a new application for a K99/R00 NIH Pathway to Independence Award from Ruth Chimenti, PhD at the University of Iowa. Dr. Chimenti proposes to investigate the mechanism-based treatment approach to chronic tendinopathy pain. The candidate has assembled a strong mentoring team with expertise in neurobiology of pain, clinical research design and statistics, led by Dr. Kathleen Sluka who has strong research support and mentoring track record. The committee agrees that the candidate has excellent clinical training in physical therapy and an outstanding track record of research productivity in biomechanics and treatment of Achilles tendinopathy with publications in high impact journals. The career development plan is well structured with clear goals to expand the research area to pain mechanisms and psychobehavioral interventions for treating chronic painful disorders. In addition, the research is focused on an under-studied clinical problem, is well-designed and innovative, and will be an excellent training vehicle for the candidate to develop scientific independence. The institutional research environment and commitment are outstanding. A few minor issues were noted in the experimental design and the statistical analysis is under developed. The remote location of the co-mentor Dr. Moseley was a concern, and it would be desirable to have an orthopedic surgeon on the mentoring team. Overall, the committee concludes the independent career developmental potential for this candidate is high.

DESCRIPTION (provided by applicant): I am a clinician-scientist committed to performing high quality research on the causes, treatment and prevention of musculoskeletal diseases. As physical therapist with PhD training in orthopaedic biomechanics, I have rigorous training in the evaluation and treatment of how people move in order to reduce tissue injury and pain. An emerging field of research indicates that changes in the central nervous system (CNS) contribute to pain in patients with orthopaedic injuries, such as tendinopathy. Yet the current standard of care for Achilles tendinopathy does not evaluate or treat CNS pain mechanisms. We propose 2 independent studies to test how altered processing within the CNS contributes to chronic Achilles tendinopathy (AT) pain. K99 phase study: Specific Aim 1.1 compares measures of altered central processing in patients with chronic AT to adults without chronic pain; Specific Aim 1.2 determines which indicators of altered central processing persist after a local anesthetic injection eliminates peripheral nociception at the site of AT pain in patients with chronic AT. R00 phase study: Specific Aim 2.1 examines if the addition of pain education to an exercise program for AT is more effective at reducing pain and disability than exercise alone using an randomized controlled trial (RCT) design; Specific Aim 2.2 determines which indicators of altered central processing are improved by the addition of pain education to an exercise intervention. The University of Iowa is a research-intensive university that facilitates my ability to obtain mentorship from leaders in the field of pain research, to collaborate with experts in clinical care and biostatistics, and to work within an academic medical center to facilitate recruitment and data collection. The short-term goals for the K99 phase of this career development award are two-fold: first, to obtain much-needed didactic coursework and experience in the evaluation of pain mechanisms; and second, is to obtain training in the design and conduct of a high quality RCT using an educational intervention in combination with exercise to treat chronic tendinopathy pain. To achieve these goals I have identified 4 key objectives: 1) Gain greater knowledge about the neurobiology of pain mechanisms; 2) Gain experience in the conduct and design of an RCT; 3) Learn about psychological factors that contribute to pain & develop a pain education intervention; and 4) Learn about and participate in statistical analysis of RCT data. The long-term goal for the R00 phase is to implement the RCT techniques learned during the K99 phase. Thus, this career development plan is designed to help me succeed in my career goal as a tenure-track professor in a top-ranked, research-intensive institution. Ultimately, I would like to perform research that can improve clinical care of individuals with chronic musculoskeletal pain, to translate this research to clinical practice, and to train future clinician-scientists.

PUBLIC HEALTH RELEVANCE: The standard of care for chronic tendinopathy primarily targets a localized area of tendon pain, but this strategy is ineffective for up to 40% of patients with chronic Achilles tendinopathy. This career development award trains a clinician-scientist to translate the latest research in pain mechanisms to revolutionize the current standard of care for tendinopathy. We propose a more global approach by evaluating and treating alterations in how the central nervous system processes chronic tendinopathy pain.

CRITIQUE 1:

Candidate: 1
Career Development Plan/Career Goals: 2
Research Plan: 3
Mentor(s), Co-Mentor(s), Consultant(s), Collaborator(s): 1
Environment Commitment to the Candidate: 1

Overall Impact: The candidate has a background in dance and physical therapy with predoctoral T32 – funded work in Linda Van Dillon's lab at Wash. U. in altered movement patterns in back pain followed by PhD studies at Rochester with Jeff Houk in clinical biomechanics and a postdoc with Marc Buckley studying insertional Achilles tendinopathy using motion analysis and ultrasound techniques. She has been extremely productive with numerous awards and publications, including 8 first-authorships, and her mentors credit her work with advancing R03 and R01 grants of their own. She has assembled an outstanding team of mentors in the neurobiology of pain, clinical research design and statistics; however the plan to integrate mentorship from Dr. Moseley raises practical (e.g. geographic) concerns and also raises the question as to whether the addition of psychologic/pain education dimensions will be truly synergistic or merely additive. The research plan is well designed to assess the role of altered CNS processing in chronic tendinopathy pain and the value of pain education, which is expected to be significant, and is innovative in quantitating the role of a local injection for non-local effects through this paradigm. Outstanding institutional environment and strong department support

1. Candidate:

Strengths

- Outstanding productivity
- Clear career path driven by curiosity and insight

Weaknesses

- None

2. Career Development Plan/Career Goals & Objectives:

Strengths

- Clear development from motion studies and biomechanics to therapeutic studies
- Neurobiology of pain natural development of disconnect between structural damage and pain perception

Weaknesses

- Leap to psychological factors/pain education less well integrated.

3. Research Plan:

Strengths

- Well designed to achieve valid conclusions
- Excellent vehicle to develop skills gained from mentorship plan

Weaknesses

- Binary outcome: how will confirmation of central contributors to pain perception specifically improve Achilles tendinopathy treatment
- Might have benefitted from teasing out interplay between motor control exercises and perturbations of the proposed biopsychosocial model (suggested by the applicant for the future)

4. Mentor(s), Co-Mentor(s), Consultant(s), Collaborator(s):

Strengths

- Dr. Sluka is an outstanding scientist with R01/UM1 funding, innovative animal model, and accomplished mentoring history including K-mechanisms.
- Dr. Rakel is an outstanding choice for RCT training; Dr. Zimmerman for statistics

Weaknesses

- Dr. Moseley (at University of South Australia) is an excellent choice to provide training and mentorship in the role of psychological factors in pain modulation and in pain education as a therapeutic strategy, but the lack of additional experts at Iowa may make integration of these skills into a research and therapy-developing program challenging.
- There is no real collaboration with orthopaedic foot and ankle surgeons except for subject referral/recruitment.

5. Environment and Institutional Commitment to the Candidate:

Strengths

- Outstanding institutional environment
- Strong departmental/Chair support

Weaknesses

- None

Protections for Human Subjects:

Acceptable Risks and Adequate Protections

Data and Safety Monitoring Plan:

Acceptable

Inclusion of Women, Minorities and Children:

- Sex/Gender: Distribution justified scientifically
- Race/Ethnicity: Distribution justified scientifically
- Inclusion/Exclusion of Children under 18: Excluding ages <18; justified scientifically

Vertebrate Animals:

Not Applicable

Biohazards:

Not Applicable

Training in the Responsible Conduct of Research:

Acceptable

Comments on Format:

- Course

Comments on Subject Matte:

- Clinical research ethics, informed consent, research misconduct, responsible authorship, COI, data integrity

Comments on Faculty Participation:

- Not specified

Comments on Duration:

- 30 hours

Comments on Frequency:

- Not specified

Resource Sharing Plans:

Acceptable

Budget and Period of Support:

Recommend as Requested

CRITIQUE 2:

Candidate: 1
Career Development Plan/Career Goals: 1
Research Plan: 5
Mentor(s), Co-Mentor(s), Consultant(s), Collaborator(s): 2
Environment Commitment to the Candidate: 1

Overall Impact: Dr. Chimenti proposes an interesting approach to an under-studied clinical problem that could be extended to other orthopaedic conditions. The described career development activities put the candidate on an excellent path to secure a faculty position at a top research institution, and be successful in conducting independent research. The main limitations are within the research plan with regard to the novel task used and statistical analysis. The strengths of the candidate and mentoring team outweigh the weaknesses of the research plan. The integration of understanding pain and analyzing movement is very important for rehabilitation research. Strong environment

1. Candidate:

Strengths

- Strong history of research productivity with publications in top journals (JOSPT, Journal of Biomechanics)
- Current funding through APTA with demonstrated history of funding
- Excellent clinical training with full time clinical experience (an asset for attaining a faculty position as a PT, PhD)
- Experiences in multiple strong research laboratories

Weaknesses

- None noted

2. Career Development Plan/Career Goals & Objectives:

Strengths

- Clearly articulated plan to obtain both knowledge and experience in selected areas of research.
- Sufficient plan to establish an independent line of research from mentors.

Weaknesses

- None noted.

3. Research Plan:

Strengths

- Aim 1 is well designed to use an anesthetic injection to test the immediate effect of pain reduction on movement, psychosocial and sensory testing.
- Pilot data is presented to support expected effect of the injection on psychosocial and sensory testing.
- The research plan includes components to facilitate the candidate's development and growth as an independent researcher.

Weaknesses

- Statistical analysis is under-developed relative to the rest of the plan.
- As written, it seems that strength and endurance will not be measured in either Aim. Given that AT is a chronic condition, and that differences in endurance have been noted following intervention, it seems that these measures would be important to include.
- The use of the waltz as a novel task is interesting. As this is used as a Movement Analysis task both pre- and post-injection (Aim 1), it would be important to consider if exposure to the task pre-injection is affecting the observed movement post-injection.
- Unclear if movement will be assessed on both sides. Assessing bilaterally would add to the understanding of the mechanisms leading to the changes in movement patterns in the presence of and reduction of unilateral pain.
- While a sub-analysis on the effect of sex is proposed for Aim 1, a similar analysis should be proposed for Aim 2, especially given the higher prevalence of AT in females.

4. Mentor(s), Co-Mentor(s), Consultant(s), Collaborator(s):

Strengths

- Appropriate mentors with strong history of productive, funded research
- Mentors at University of Iowa have a history of collaboration.

Weaknesses

- Dr. Moseley is not on-site (minor weakness)

5. Environment and Institutional Commitment to the Candidate:

Strengths

- Strong environment for K99 components
- Well positioned to secure appropriate R00 position.

Weaknesses

- None noted.

Protections for Human Subjects:

Acceptable Risks and Adequate Protections

Data and Safety Monitoring Plan:

Unacceptable

- Does not seem well developed for the proposed clinical trial in Aim 2.

Inclusion of Women, Minorities and Children:

- Sex/Gender: Distribution justified scientifically
- Race/Ethnicity: Distribution justified scientifically
- Inclusion/Exclusion of Children under 18: Including ages < 18; justified scientifically

Training in the Responsible Conduct of Research:

Acceptable

Comments on Format:

- Addressed and appropriate

Comments on Subject Matter:

- Addressed and appropriate

Comments on Faculty Participation:

- Addressed and appropriate

Comments on Duration:

- Addressed and appropriate

Comments on Frequency:

- Addressed and appropriate

Budget and Period of Support:

Recommend as Requested

CRITIQUE 3:

Candidate: 2
Career Development Plan/Career Goals: 1
Research Plan: 2
Mentor(s), Co-Mentor(s), Consultant(s), Collaborator(s): 1
Environment Commitment to the Candidate: 1

Overall Impact: This is an outstanding transition to independence application from a well-trained and accomplished young investigator. The PI has done excellent work in the biomechanics and treatment of Achilles tendinopathy and is expanding her expertise and research program into a broader view of pain mechanisms and psychobehavioral interventions for treating chronic painful disorders. Her mentors are highly accomplished researchers and research mentors who are well positioned to assist the candidate's transition to independence. The training plan, working of different aspects of her mentors ongoing projects with subsequent translation into her own proposed pilot study is well designed and should prepare the applicant for a successful transition. The proposed study is well designed, innovative and significant. It is an excellent vehicle for the applicant's career development. The research environment is outstanding.

Protections for Human Subjects:

Acceptable Risks and Adequate Protections

Inclusion of Women, Minorities and Children:

- Sex/Gender: Distribution justified scientifically
- Race/Ethnicity: Distribution justified scientifically
- Inclusion/Exclusion of Children under 18: Excluding ages <18; justified scientifically

Training in the Responsible Conduct of Research:

Acceptable

Budget and Period of Support:

Recommend as Requested

THE FOLLOWING SECTIONS WERE PREPARED BY THE SCIENTIFIC REVIEW OFFICER TO SUMMARIZE THE OUTCOME OF DISCUSSIONS OF THE REVIEW COMMITTEE, OR REVIEWERS' WRITTEN CRITIQUES, ON THE FOLLOWING ISSUES:

PROTECTION OF HUMAN SUBJECTS (Resume): ACCEPTABLE
Human subjects are involved; there are no concerns.

INCLUSION OF WOMEN PLAN (Resume): ACCEPTABLE
Women and men will be part of the study; scientifically this is acceptable.

INCLUSION OF MINORITIES PLAN (Resume): ACCEPTABLE

Minorities and non-minorities are involved in the proposed project; their participation appears appropriate.

INCLUSION OF CHILDREN PLAN (Resume): ACCEPTABLE
Only adults will be part of the study; scientifically this is acceptable.

SCIENTIFIC REVIEW OFFICER'S NOTE: A clinical trial is proposed in Aim2 and the application does not have a well-developed Data and Safety Monitoring Plan.

TRAINING IN THE RESPONSIBLE CONDUCT OF RESEARCH: ACCEPTABLE

COMMITTEE BUDGET RECOMMENDATIONS: The budget was recommended as requested.

Footnotes for 1 K99 AR071517-01; PI Name: Chimenti, Ruth Louise Porter

NIH has modified its policy regarding the receipt of resubmissions (amended applications). See Guide Notice NOT-OD-14-074 at http://grants.nih.gov/grants/guide/notice-files/NOT-OD-14-074.html. The impact/priority score is calculated after discussion of an application by averaging the overall scores (1-9) given by all voting reviewers on the committee and multiplying by 10. The criterion scores are submitted prior to the meeting by the individual reviewers assigned to an application, and are not discussed specifically at the review meeting or calculated into the overall impact score. Some applications also receive a percentile ranking. For details on the review process, see http://grants.nih.gov/grants/peer_review_process.htm#scoring.

MEETING ROSTER
Arthritis and Musculoskeletal and Skin Diseases Special Grants Review Committee
Arthritis and Musculoskeletal and Skin Diseases Initial Review Group
NATIONAL INSTITUTE OF ARTHRITIS AND MUSCULOSKELETAL AND SKIN DISEASES

AMS
11/03/2016 - 11/04/2016

CHAIRPERSON(S)

LEE, DELPHINE J, PHD, MD
CHIEF
DIVISION OF DERMATOLOGY
DEPARTMENT OF MEDICINE
LOS ANGELES BIOMEDICAL RESEARCH INSTITUTE/
HARBOR-UCLA MEDICAL CENTER
TORRANCE, CA 90502

MEMBERS

ADAMS, JOHN S, MD
PROFESSOR
DEPARTMENT OF ORTHOPAEDIC SURGERY
AND MOLECULAR, CELL AND DEVELOPMENTAL BIOLOGY
UNIVERSITY OF CALIFORNIA, LOS ANGELES
LOS ANGELES, CA 90095

AHMED, SALAH-UDDIN, PHD *
ASSOCIATE PROFESSOR
DEPARTMENT OF PHARMACEUTICAL SCIENCES
COLLEGE OF PHARMACY
WASHINGTON STATE UNIVERSITY
SPOKANE, WA 99204

ANDRADE, FRANCISCO H, PHD
PROFESSOR
DEPARTMENT OF PHYSIOLOGY
UNIVERSITY OF KENTUCKY
LEXINGTON, KY 40536

BAAR, KEITH, PHD *
ASSOCIATE PROFESSOR
DEPARTMENT OF PHYSIOLOGY AND MEMBRANE BIOLOGY
SCHOOL OF MEDICINE
UNIVERSITY OF CALIFORNIA DAVIS
DAVIS, CA 95616

BURR, DAVID B, PHD
PROFESSOR
DEPARTMENT OF ANATOMY AND CELL BIOLOGY
INDIANA UNIVERSITY SCHOOL OF MEDICINE
INDIANAPOLIS, IN 46202

CLAUW, DANIEL J, MD *
PROFESSOR OF ANESTHESIOLOGY, MEDICINE AND
PSYCHIATRY
DIVISION OF ANESTESIOLOGY
UNIVERSITY OF MICHIGAN
ANN ARBOR, MI 48105

COOPER, KEVIN D, MD
PROFESSOR AND CHAIRMAN
DEPARTMENT OF DERMATOLOGY
CASE WESTERN RESERVE UNIVERSITY
CLEVELAND, OH 44106

COSTENBADER, KAREN H, MD
ASSOCIATE PROFESSOR
DIVISION OF RHEUMATOLOGY, IMMUNOLOGY AND
ALLERGY
BRIGHAM AND WOMEN'S HOSPITAL
BOSTON, MA 02115

CROFFORD, LESLIE J, MD
PROFESSOR OF MEDICINE
DIVISION OF RHEUMATOLOGY AND IMMUNOLOGY
VANDERBUILT UNIVERSITY SCHOOL OF MEDICINE
NASHVILLE, TN 37232

DIRKSEN, ROBERT T, PHD
LEWIS PRATT ROSS PROFESSOR
DEPARTMENT OF PHARMACOLOGY AND PHYSIOLOGY
SCHOOL OF MEDICINE AND DENTISTRY
UNIVERSITY OF ROCHESTER
ROCHESTER, NY 14642

FEGHALI-BOSTWICK, CAROL A, PHD *
KITTY TRASK HOLT ENDOWED CHAIR
DIVISION OF RHEUMATOLOGY AND IMMUNOLOGY
DEPARTMENT OF MEDICINE
MEDICAL UNIVERSITY OF SOUTH CAROLINA
CHARLESTON, SC 29425

FLATOW, EVAN L, MD
PROFESSOR
DEPARTMENT OF ORTHOPAEDIC SURGERY
MOUNT SINAI SCHOOL OF MEDICINE
NEW YORK, NY 10029

FLUM, DAVID R, MD *
PROFESSOR AND ASSOCIATE CHAIR
DEPARTMENT OF SURGERY
SCHOOL OF MEDICINE
UNIVERSITY OF WASHINGTON
SEATTLE, WA 98195

FRAENKEL, LIANA, MPH, MD
ASSOCIATE PROFESSOR OF MEDICINE
DEPARTMENT OF MEDICINE
YALE UNIVERSITY SCHOOL OF MEDICINE
NEW HAVEN, CT 06520

GARZA, LUIS ANDRES, PHD, MD
ASSOCIATE PROFESSOR
DEPARTMENT OF DERMATOLOGY
JOHNS HOPKINS UNIVERSITY
BALTIMORE, MD 21287

KIM, BRIAN, MD *
ASSISTANT PROFESSOR
DIVISION OF DERMATOLOGY
DEPARTMENT OF INTERNAL MEDICINE
WASHINGTON UNIVERSITY
ST. LOUIS, MO, MO 63110

KOCH, PETER J., PHD *
PROFESSOR
CHARLES C GATES CENTER FOR REGENERATIVE
MEDICINE AND STEM CELL BIOLOGY
DEPARTMENT OF DERMATOLOGY
UNIVERSITY OF COLORADO, DENVER
AURORA, CO 80045

LANE, JOSEPH MICHAEL, MD *
PROFESSOR AND ASSISTANT DEAN
DEPARTMENT OF SURGERY
HOSPITAL FOR SPECIAL SURGERY
NEW YORK, NY 10021

LEWIS, CARA L, PHD *
ASSISTANT PROFESSOR
CLINICAL EPIDEMIOLOGY RESEARCH AND TRAINING UNIT
DEPARTMENT OF MEDICINE
BOSTON UNIVERSITY SCHOOL OF MEDICINE
BOSTON, MA 02215

LURIE, JON D, MD
ASSOCIATE PROFESSOR
DEPARTMENT OF ORTHOPAEDICS
DARTMOUTH HITCHCOCK MEDICAL CENTER
DARTMOUTH COLLEGE
LEBANON, NH 03756

MAHONEY, MY GEORGIA, PHD
PROFESSOR
DEPARTMENT OF DERMATOLOGY AND CUTANEOUS
BIOLOGY
AND BIOCHEMISTRY AND MOLECULAR BIOLOGY
JEFFERSON MEDICAL COLLEGE
THOMAS JEFFERSON UNIVERSITY
PHILADELPHIA, PA 19107

MI, QING-SHENG, PHD, MD *
PROFESSOR
HENRY FORD IMMUNOLOGY PROGRAM
DEPT OF INTERNAL MEDICINE
HENRY FORD HEALTH SYSTEM
DETROIT, MI 48202

PHAM, CHRISTINE T, MD
ASSOCIATE PROFESSOR
DEPARTMENT OF RHEUMATOLOGY
WASHINGTON UNIVERSITY
ST LOUIS, MO 63110

QIN, YI-XIAN, PHD
PROFESSOR
DEPARTMENT OF BIOMEDICAL ENGINEERING
STATE UNIVERSITY OF NEW YORK AT STONY BROOK
STONY BROOK, NY 11794

THOMPSON, SUSAN D, PHD *
PROFESSOR
CINCINNATI CHILDRENS HOSPITAL MEDICAL CENTER
CENTER FOR AUTOIMMUNE GENOMICS AND ETIOLOGY
UNIVERSITY OF CINCINNATI COLLEGE OF MEDICINE
CINCINNATI, OH 45229

WILLIAMS, BART O, PHD
PROFESSOR AND DIRECTOR
CENTER FOR CANCER AND CELL BIOLOGY
VAN ANDEL RESEARCH INSTITUTE
GRAND RAPIDS, MI 49503

SCIENTIFIC REVIEW OFFICER

LIN, HELEN, PHD
SCIENTIFIC REVIEW OFFICER
NATIONAL INSTITUTES OF HEALTH
NATIONAL INSTITUTE OF ARTHRITIS,
MUSCULOSKELETAL, AND SKIN DISEASES
BETHESDA, MD 20892

EXTRAMURAL SUPPORT ASSISTANT

SAVAGE, HANNAH M
EXTRAMURAL SUPPORT ASSISTANT
NATIONAL INSTITUTE OF ARTHRITIS,
AND MUSCULOSKELETAL AND SKIN DISEASES
NATIONAL INSTITUTES OF HEALTH
BETHESDA, MD 20892

UDDIN, HUMERA SHARIF
EXTRAMURAL SUPPORT ASSISTANT
SCIENTIFIC REVIEW BRANCH
NATIONAL INSTITUTE OF ARTHRITIS
AND MUSCUKOSKELETAL AND SKIN DISEASES
NATIONAL INSTITUTES OF HEALTH
BETHESDA, MD 20892

* Temporary Member. For grant applications, temporary members may participate in the entire meeting or may review only selected applications as needed.

Consultants are required to absent themselves from the room during the review of any application if their presence would constitute or appear to constitute a conflict of interest.
